# Do-it-yourself biology and electronic waste hacking: A politics of demonstration in precarious times

**DOI:** 10.1177/0963662516647348

**Published:** 2016-08-02

**Authors:** Ana Delgado, Blanca Callén

**Affiliations:** University of Bergen, Norway; BAU Design College of Barcelona, Spain

**Keywords:** governance of science and technology, interaction experts/publics, scientific citizenship, social movements

## Abstract

In recent years, there has been an explosion of do it yourself, maker and hacker spaces in Europe. Through makers and do-it-yourself initiatives, ‘hacking’ is moving into the everyday life of citizens. This article explores the collective and political nature of those hacks by reporting on empirical work on electronic waste and do-it-yourself biology hacking. Using Dewey’s experimental approach to politics, we analyse hacks as ‘inquiry’ to see how they serve to articulate public and political action. We argue that do-it-yourself and makers’ hacks are technical and political demonstrations. What do-it-yourself and makers’ hacks ultimately demonstrate is that things can be done otherwise and that ‘you’ can also do it. In this sense, they have a potential viral effect. The final part of the article explores some potential shortcomings of such politics of demonstration.

## 1. Introduction: Hacking as demonstrating

This article looks at DIYbio (do-it-yourself biology) and electronic waste (e-waste) hacking and suggests that hacks work as demonstrations in both a technical and political sense. Hackerspaces, medialabs, Fablabs and open labs are rapidly popping up all over the globe, transferring the practice of hacking and the ethos of sharing (Delfanti, 2013) into mundane domains of citizens’ everyday lives. Practices of ‘DIY Medicine’, for instance, are becoming quite common (Dolgin, 2010), enabling production of low-cost and portable technologies, such as simple sensors and genetic tests, allowing non-scientist citizens to check themselves for genetic diseases. Such DIY devices are often ‘open’ in that they come accompanied by instructions on how they were made, making it possible for others to replicate, modify and develop them. They are objects to be shared, often called ‘hacks’ by their makers. In the technical visibility that they display, they appear as demonstrations. However, they are also demonstrations in a political sense. We note that DIY and makers’ hacks are spreading at a time in which public health coverage is being withdrawn in many countries and private health services are unaffordable for many – a time in which the body is increasingly experienced as a precarious and vulnerable entity. DIY medical devices can be seen as revealing such precariousness while showing that it is possible for citizens to find technical ways to cope with it.

The rise of DIY (Levine and Heimerl, 2008; Spencer, 2008) has received attention in many scholarly works, which have offered a colourful picture. Some attend to its aesthetic, leisure and creative dimensions (Roosth, 2010; Walter-Herrmann and Büching, 2013); some focus on the empowering effects of such practices for the citizenry (Atkinson, 2006; Petersen, 2014; Proulx, 2009; Ratto and Boler, 2014); and others emphasise how DIY challenges conventional producer–consumer relations (Campbell, 2005; Shove et al., 2007; Watson and Shove, 2008). Finally, some works suggest that the hacking culture can be seen as an innovative element within the neoliberal dynamics of capitalism (Söderberg and Delfanti, 2015). This article takes a different angle: it situates DIY and makers’ practices in a time of ‘precarity’, which is understood as the loss of social rights and material securities (Butler, 2009). Seen in this light, we ask *how might DIY and makers’ hacks be political actions? How do they claim things as public?*

In addressing our questions, we provide insights from the authors’ recent ethnographic fieldwork^1^ on DIYbio and e-waste hacking in Europe.^2^ As we will show, both DIYbio and e-waste hacking emerge in an ambiguous relation with ‘precariousness’ (and ‘precarity’).^3^ They are a product and a reaction to a situation of economic, social and material shakiness, deriving from overproduction and rapid technological replacement. E-waste hacking emerges as a response to the designed obsolescence of electronic devices and operates by repairing and reusing discarded and obviated technologies. Waste is the object of hacks as well as the material condition of their possibility. Similarly, DIYbio has originated in a situation of technological excess and speed that combines a rapid drop in the price of technologies, such as gene sequencing and synthesising (Carlson, 2010), with the recurrence of bankruptcy and crisis in Big Bio business. As a consequence of both, cheap and second-hand lab equipment has become available on sites such as eBay, opening the possibility of setting up a lab for less than a US$1000 (Wohlsen, 2011). This sort of ‘low-cost’ biology is also a product of a knowledge-based economy that produces a surplus of technically literate (young) people who might not suit the type of jobs that an increasingly precarious labour market can offer (Neilson and Rossiter, 2005).

In our approach to DIYbio and e-waste hacking, we find inspiration in John Dewey’s (1927) ‘*The Public and its problems. An essay on Political Inquiry*’. We recall attention to the ‘inquiry’ as a possible means for experimental politics. In Dewey’s work, political action resides in the very moment of the emergence of a public, when heterogeneous sectors of the citizenry gather together as a temporary collectivity around an issue affecting them (Marres, 2007). Our focus in this article, however, is not on how the public is triggered into being (not whether DIY and makers are publics in a Deweyan sense), but on how things are claimed as public through particular forms of collective action, in this case, hacking.

The structure of the article follows this argument: DIYbio and e-waste hacking is a product and a response to the *current state of things*. As instances of collective inquiry (Dewey, 2008 [1938]), hacks disclose objects, showing them to be undetermined and modifiable (Callén, under review; Delgado, 2013). By making visible the ontological precariousness of realities, they open up the possibility for political action (Butler, 2004). DIY and makers’ hacks are political demonstrations in that they tangibly *show* that realities can be modified and that ‘*you*’ can do it. They cope with a situation of increasing precarisation by playing on the precariousness of objects. Paradoxically, we argue towards the conclusion of the article that this might end up having a non-desired effect if DIY political action results in the acceptance of political precarity.

## 2. Hacking precarity from inside the welfare-state ruins

As early as 1927, Dewey expressed concern with the development of industrialisation, consumer societies and the shortcomings of representative mechanisms in a liberal democracy. In a pragmatist approach to politics, he pays special attention to the common material conditions of life. Our arguments in this article are driven by similar concerns: the precarisation of work conditions and social security infrastructures has been a common trend in European countries, even before the financial crisis that started in 2008. While these trends accelerate, the European Commission keeps referring to an imagined ‘Innovation Union’ (Barroso, 2011) in which citizens ought to take an active role in addressing ‘grand challenges’, such as climate change and ageing. Such technical activation of the citizenry might appear as a remedy to cope with a double crisis: an economic one and one of political representativeness.

In his analysis, Dewey emphasised the development of communication technologies, such as phones, telegraphs and roads, as well as industry, as creating the kinds of problems affecting citizens. Currently, in a kind of parallelism, European citizens are strongly affected by the extended consequences of decaying welfare infrastructures and emergence of new infrastructures supporting knowledge-based and innovation-driven economies, such as those arising through the Internet. These consequences have triggered public concern of a different sort around property, access and social agency; in addition, information and communications technologies (ICTs) clearly shape the process of issue articulation as well. We see that through the use of digital technologies, sectors of the citizenry are increasingly self-organising around issues connected to areas such as health and care (Akrich et al., 2009; Brown et al., 2004), urbanism (Corsín and Estalella, 2013; Estalella, 2014) and education (Kamenetz, 2010), where public infrastructures tend to collapse. Such initiatives are diverse, some initiated by young and technically literate people who explicitly use the labels of ‘DIY’ and ‘hacking’ to define them. However, how might those technical interventions be public and political action? According to Dewey, through deliberation, issues of common concern should in healthy democracies be articulated within experimental processes. This is a creative and exploratory action: a sort of collective inquiry. At the core of this creative moment, Dewey postulates the imagination, understood as the capacity to understand the actual in light of the possible. Dewey’s inquiry is a form of knowledge-making that is intrinsic to political action. Practised as an exploratory action, political action is the possibility of breaking with established habits and orders that have become problematic and experimentally opening up to the emergence of new or unforeseen possibilities for life in common.

Although DIY and makers’ hacks may in some ways appear as an ‘inquiry’, as we will see, interpreting them as public action from a Deweyan approach is problematic. Even when the public gathers around an issue of common concern, in a second step, when public issues have become visible and pressing, the State should develop some means to deal with them (Dewey, 1927). Democratic institutions are put in place for that purpose. In this account, the State ultimately makes sense as an adjudicator of public problems. Publics and experts, especially state officials, have separate roles in this approach. However, what happens when citizens do not expect the State to take care of their issues? How are these issues dealt with as public? By presenting empirical materials on hacking, the next section suggests a move from a politics of representation to a politics of demonstration.

## 3. Hacking life and waste

### DIYbio

DIYbio is a global network of heterogeneous local groups that develop ‘hacks’ of different sorts on living matter. These technological interventions are presented as citizen science, art or a new way of approaching business based on open-source practices (or sometimes a combination). In view of such diversity, some scholars have pointed to the difficulties in situating DIYbio hackers as liberals or libertarians, activists or entrepreneurs (Delfanti, 2011; Kelty, 2010). Despite such kaleidoscopic diversity, DIYbio groups in Europe and elsewhere have invested much work and time in organising knowledge-sharing activities, such as workshops.

**Figure 1.**
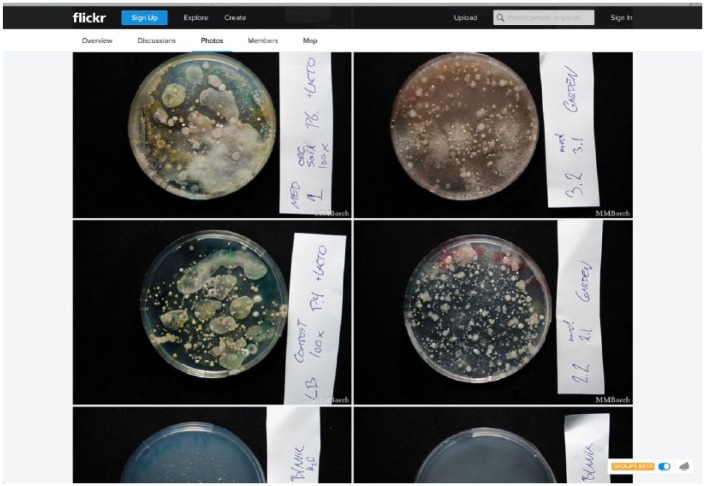
BioStrike results documented in Flirck: https://www.flickr.com/groups/biostrike/

This section of the article brings the reader to the situation of one of these workshops that took place in the fall of 2014 at a public university in Germany. With approximately 15 participants, the session was designed to show people how to ‘hack’ the problem of increasing bacterial resistance to antibiotics, presented as a public health problem. There were two tables in the room with a mixture of basic lab equipment (such as Petri dishes, pipettes and different sorts of solutions) and some kitchen equipment and utensils (a pressure cooker, a hotplate and tooth picks, among other things). The group was split into two. With the guidance of an instructor, part of the group performed DNA extractions from local soil samplers. Then, they were introduced to the process of ‘horizontal gene transfer’ key to the spreading of antibiotic resistance in the environment. Another instructor asked the rest of us to search for samples in our immediate surroundings. Diverse types of things were picked by the participants as possible sites of antibiotic resistance. One participant suggested to an author of the article that she extracted samples from the inner ears and nostrils. As participants put together a collection of samples, the cooker was boiling with a mixture of beef stock and milk powder, creating a home-made ‘broth’, the medium to fill the Petri dishes into which the samples would be inserted. Professional labs buy kits with standard versions of this medium, which are expected to add reliability and stability to the experiment. However, making the broth is a way of showing that performing biological experiments is accessible. The broth was transferred to the Pedri dishes and the samplers were divided among them and pre-bought Petri dishes containing antibiotics at different concentrations. We waited to see whether something would grow in the Petri dishes. Ultimately, the absence of ‘zones of inhibition’ (areas where nothing grows; Figure 1) would effectively *show* the growth of antibiotic resistance.

Arguably, in the BioStrike project, the object of the hack is manifold: the monopolies of the pharmaceutical industry, individual bodies, a shared environment, tools and procedures, and also the audience’s view on the biological world. Hacking thus appears as the action of opening those multiple sites for exploration, perhaps for modification. By using simple methods, unconventional tools (working in a ‘use what is there’ mode), approximated metrics, non-standardized mediums and a non-systematic data collection, we were shown that doing biology is easy and accessible and that *you can also do it*. That was the message that this hack seems to carry. Although participants did not necessarily think that the experiment was as simple as to be replicable at home, most did think that the session was fun and engaging.^4^

This workshop was one of the BioStrike project series that has been touring Europe since 2012. It was uncommon insofar as it took place at an institution, the public university Karlsruhe Institute of Technology. Usual sites for the BioStrike workshops have been hacker spaces, art festivals and other public venues. Attendees of this event were in a way special too because they were mostly social scientists and philosophers of science. In contrast, these series of workshops often have more diverse audiences, including artists and ‘just citizens’. Despite those variations, the format of the workshop was akin to those done before in different European cities. DIYbio Europe groups have joined efforts to ensure such replicability through collective sharing, performed in the design of the BioStrike experiments and workshops. The protocols, mediums, tools and materials needed for the experiments and the experiences of others while running the experiment are all documented and accessible in a number of online platforms, such as wikis, Flickr, Google drive documents, Google group discussions and open lab books.^5^ The purpose of such extensive sharing and circulation (Meyer, 2012) is that others can replicate, even improve the hack.

As documented in those online sites, in the complete version of the BioStrike experiment, identification of antibiotic resistance is to be complemented with discovery of natural sources of antibiotic substances produced by wild bacteria. This end stage to the ‘hack’ concerns the production of antibiotics and is presented as a way to ‘hack’ the pharmaceutical industry. In this step, citizens (globally ideally) would participate in a massive bioprospecting^6^ process. Images of dishes showing ‘zones of inhibition’ of antibiotic resistance would be shared (using an online platform) for a massive screening of the best antibiotic producer (Figure 1). Using the Internet as a platform for display, all the technical details of this ‘citizen science’ crowd-sourced experiment would be shared and made visible to all; this visibility would itself be proof that the problem could be tackled at a low cost and in accessible ways through cooperative and decentralised work.

**Figure 2.**
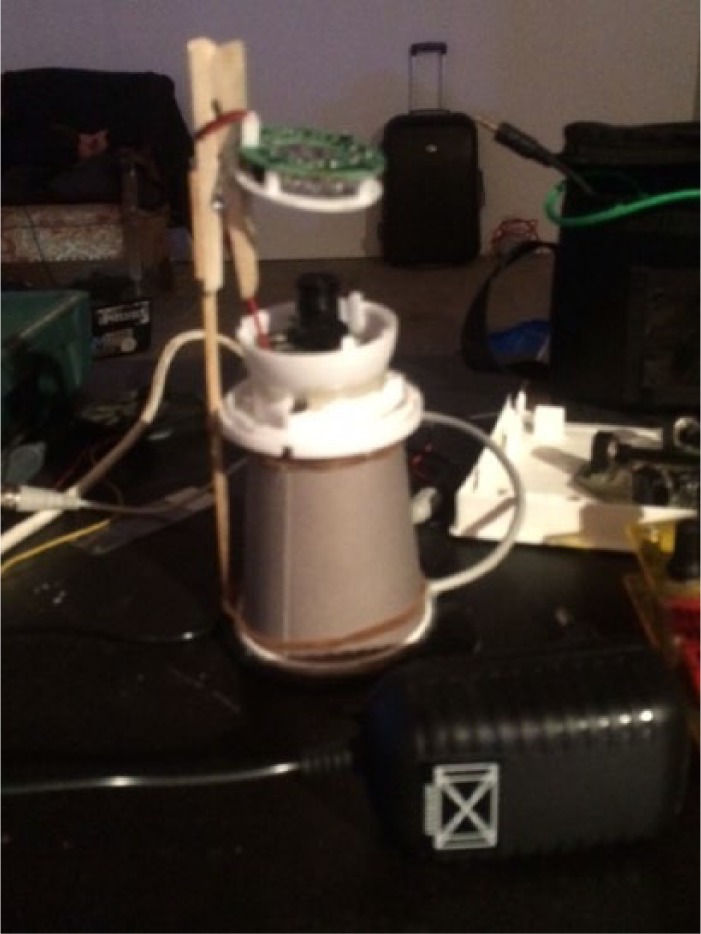
Microscope made with disposable coffee cup and clothing clip. Picture taken at Piksel workshop by the author in Bergen (Norway), November 2014.

Rather than providing a definitive solution for the global problem of antibiotic resistance, the BioStrike project is better understood as a site for learning, an ongoing experiment and a collective exercise of inquiry. In DIYbio environments, it is often emphasised that whether a hack works is not as important as just doing it: figure out the problem, see what you need and what you can get, be creative and use your surroundings (Figure 2).

One the most interesting aspects of the BioStrike project is that it has created room for a debate on the issue of the ‘biocommons’, raising visibility on illicit appropriations on life. Such concern is emphasised by ‘Hackteria’, a global DIYBio network originated in Zurich, when claiming that ‘We have always been biohackers’. With this slogan, they reclaim mundane activities, such as cooking as forms of hacking. In reimaging biology as mundane, public access to it is claimed, as another DIYbio practitioner based in Copenhagen explained:

Biology has been deemed quite holy, that you cannot manipulate biology or if you do that you are playing God. I would like to inform people and to remove that kind of preconception. Biology has always been changing and always manipulating itself: that is the evolutionary, dynamic process. But there is a big fear. And because of that (fear), universities and big pharmaceutical companies end up keeping a monopoly to develop and change biology […] I would like to be able to communicate that that is not the case, that biology has always been used by people, we are biology and biology changes by itself. To be able to get that point out is kind of the first step. So people realize that they can do it, and they can use biology in another way than just getting pre-packaged solutions that has been stamped by a university or a big company. The next step, another point, is citizen science being the knowledge sharing between the communities that actually use it. (DIYbio practitioner, Copenhagen 2012)

Some DIYbio groups in Europe have directed their efforts to developing low-cost and portable lab tools that will allow others to ‘mess with’ the biological world. The Bento lab,^7^ a modular and portable DNA analysis laboratory that includes a polymerase chain reaction (PCR) machine, a centrifuge and a gel electrophoresis box, is an instance of this. In addition to producing accessible tools, some groups produce innovative and low-cost market products such as the organic ink produced by La Paillasse, the local group in Paris and Lyon (based on soil bacteria and activated by using the acid of a lemon). For commercial projects such as this, a requirement is often that the product be developed ‘open source’, meaning that blueprints are available and materials accessible to enable ‘others’ to replicate and improve on the original. Open source appears as a new way of producing value that emerges from the experimental and prototyping nature of DIYbio designs. Although some emphasise the ‘economic’ and others the ‘social’, value is seen by all as produced in the wide circulation of biological designs.

The precarious dimension of DIY and open-source tinkering is often emphasised in the literature as holding creative potential (Delgado, 2013; Kera, 2014). What is rarely emphasised is precarity as the material context of existence of the DIY scene. In a recent public meeting in La Paillasse, this hacker space was presented as an ‘opportunity’ for young scientists to perform biology more freely than is commonly possible in academic settings (namely, by engaging in more creative projects with less standard methods). La Paillasse is one of the most established groups in Europe. They recently got a good deal with the City Council in Paris and are allowed to use (and for that matter, also maintain) an old building right in the city centre at a relatively low cost. They rent out part of this large room to get some funding for their projects, combining this solution with micro-funding sources. A power point slide was shown during the presentation in La Paillasse that pointed to what seemed to be a worrying situation: the average age of a researcher in biology receiving a first grant is 40 years. The lack of opportunities for young researchers is something one hears mentioned often within DIYbio environments. Job precarisation, the dominance of hierarchies and high pressure for publishing are indeed recurrent elements in universities and biotech industry environments. That is especially so for young researchers. In this context, one alternative is hacking biology, that is, turning it into a low-cost and creative technical practice. This is done by producing a way of relating to biology and technology that is not delegated but ‘owned’ (not necessarily in a proprietary sense, but in the sense of ‘appropriated’). As Hackteria wrote in its wiki during the Piksel Festival in Bergen in 2014, ‘If you can’t build your Lab you don’t *own* the Lab’.

### Obsoletos: hacking e-waste

In this section, we present Obsoletos (http://obsoletos.org/), a small-scale hacker and maker initiative in Madrid, composed originally of four friends who were trained in different scientific and technical disciplines. In their own words, their work is ‘driven by their curiosity’ to see what can be made out of e-waste. At the core of their activities, they perform ‘hacks’ from discarded components and devices; they document them in a blog where they also discuss different aspects of technological obsolescence. For a couple of years, Obsoletos also organised workshops and meetings to train others in how to rebuild old computers or to hack and create new devices from recovered e-waste components. These workshops were often run in public universities and cultural institutions, with financial support from the Spanish Ministry of Culture. Although Obsoletos ran out of funding for this type of activity a few years ago, they still update their blog, collaborate with other groups and keep developing new hacks and creations, such as a three-dimensional (3D) laser-cutter-printer that became a shared tool in La Nave, a co-working space where they can partake cheaply with other maker groups.

E-waste, the object of Obsoletos’ hacks and creations, is seen by them as the harmful and hidden material effect of the ethereal promises and imaginaries of progress and innovation associated with electronics and the information and knowledge in society. Constant software updating and the accelerating escalation of hardware replacement by more powerful devices in a ‘throw away’ society promote unsustainable consumption patterns with two important effects. First, it aggravates the digital divide by making it more difficult and expensive for many people to participate and keep up with the information society. Second, e-waste has hazardous and polluting effects on environments and the health of those who work on treating and recycling the irregularly exported e-waste. Obsoletos explicitly acknowledged that their workshops and hacks might not solve the problem of overproduction of e-waste due to their minor scale of action, but it might attract attention to such a problem: ‘The real and direct impact is zero. Our aim is that people see it and say, ‘Oh! Here, there may be a problem’. We want to make it visible rather than solving anything’.

**Figure 3.**
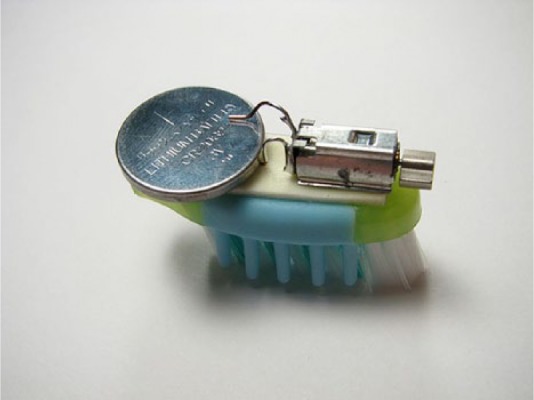
By Obsoletos, CC BY-SA 3.0 ES licensing.

By performing and sharing simple hacks on their blog, they display e-waste as a site that can be disclosed and intervened through fun and creative actions but also as a mundane and common problem. On the blog, there are many descriptions on how hacks were performed. Here, we focus on the BristleBot, an amusing ‘robotic creature’ that moves and is powered by a button battery attached to a small vibrator motor recovered from an obsolete mobile phone, added to a bristle or a chip (Figure 3).

**Figure 4.**
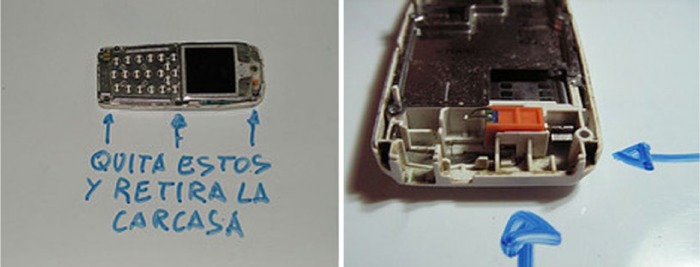
By Obsoletos with CC BY-SA 3.0 ES licensing. The written instruction says, ‘unscrew these and remove the cover’.

One of Obsoletos’ members explains how he extracted the vibrator motor out of a couple of old Nokia mobile phones he had at home: after unscrewing the torx screws from the phone case where the circuit board is attached, the board was removed because just behind it, next to the power outlet, is where the ‘famous’ vibrator is located (Figure 4). Not welded or glued, it can be removed directly. Once the vibrator is extracted, both contacts have to be bent so that one touches the button battery while the other remains free so it can be connected to the bristle using double-sided sticky tape.

**Figure 5.**
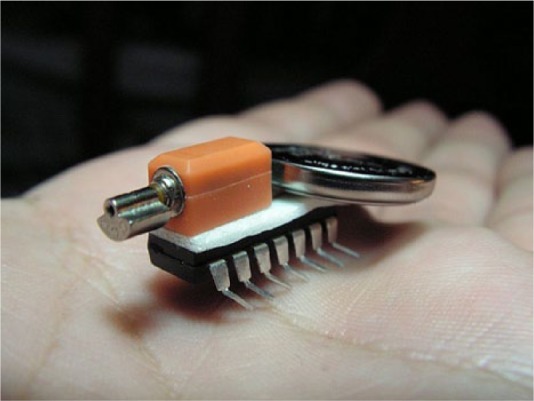
By Obsoletos with CC BY-SA 3.0 ES licensing.

The Obsoletos’ hacker could not find a toothbrush with ‘collaborative’ bristles. However, he was inspired by a picture that somebody had shared on an Internet site called ‘Evil Mad Scientist’, so turned the initial idea of the BristleBot into a ‘ChipBot’ (Figure 5). This kind of chip, as he explains, is easy to find because they are included almost in anything that plugs in or works with batteries and anything that ‘you were almost (certainly) going to throw away’.

The appeal of using common and ‘very easy to find’ discarded objects as resources, together with the pedagogical and direct tone of the descriptions in the blog, is that they are a powerful means to popularise hacks and try to prove that ‘you’, or anyone, can make them. Even when defining ‘hacking’ on their blogsite, they draw on a popular parallelism with their mothers’ cooking:

In the same way that she adapts the recipes of Arguiñano (a popular Spanish cook) for making up for the lack of some ingredient, we adapt and reuse pieces of computers. My mother feels fulfilled with her food and even though she doesn’t even think about it, what she does shows that she knows the rules sufficiently as to be able to break them. She is a hacker of the kitchen. […] We do want to know how things work. And once we know how they work, the typical result is to realise that they could work a bit better.

A manifesto published in Obsoletos’ blog tells the reader what hacking is:

Isn’t the phenomena of hacking present everywhere and at any time? Effectively, hacking is everywhere. As long as we live in a world with established rules, there will be hackers. Hacking is the experimental modification of systems out of creativity or for obtaining advantages (Obsoletos.org, 2008).

Thus, they reclaim the creative empowerment of hacking against ‘a post-industrial alienation’ where everything has been designed by someone different from the users, given the role of mere consumers. Their argument is that modifying the systems around them gives them practical advantages and control. By hacking, they say, ‘you gain power over the objects’.

In practice, their hacking method is simple. As we saw for the chip-bot, they have raw material in the shape of e-waste around them and ask themselves, ‘What can we do with it that is cool?’ Guided by enjoyment, they search on the Internet for ideas and just start doing them. Then, ‘You try, you expand the idea or you just mix and combine things with other things until it turns into something that works’, they explained during the interviews. They also advise not to despair and to give it a couple of tries before leaving it alone for a while. Many of Obsoletos’ hacks start with a prototype which is improved afterwards. Their workshops’ goal is not to produce a ready-made and finished object but to provide some tools so that people can use them in their own projects. To spread these hacks and facilitate the appropriation of such tools, documentation and sharing are crucial. Obsoletos carefully document their hacks on their blog and express their gratitude to other unknown people and online communities who do the same: ‘You make and you give back’, they say. Basically, ‘what happens to you has usually also happened to someone else’, and in a kind of a spreading fashion, ‘all the hacks come in a sense from what you have seen somewhere else’.

Throughout this process, by refurbishing and hacking obsolete computers or any kind of electronics to transform them into new devices and inventions, such as a soap bubble-maker, hard drive speaker or laser oscilloscope, Obsoletos’ hackers bring to light the unintended side effects of our supposedly dematerialised ‘digital culture’. They attract attention to an issue and confront it strategically, namely, the obsolescence and precariousness produced by economic cycles of design, manufacturing, consumption and disposal of technological innovations. However, they say, they do not want to ‘preach’, nor do they want to call upon a collective or set up a group to address those issues in the way that political activism is often performed. As they explain about their workshops and blogsite documentation, they just want to make available all the possible information they have collected so ‘that anyone who wants to could do it at home just as we do’. Obsoletos want people to try on their own and afterwards send a message back and to say ‘look what I’ve made!’ They are interested in the replicability of things and showing that anybody can experiment with e-waste if they so desire. In summary, as one of the workshops’ assistants said about Obsoletos,

The interesting thing is *to know the broad possibilities that, with a bit of imagination and those resources, we all have*. However, nobody wants them^8^ because they are considered to be obsolete. We can change our perspective to see something useless as a new device with a totally different end or to aim at restoring the use it previously had. (Emphasis added)

## 4. An inquiry into objects: Making issues public

The empirical sections above show how by hacking biological practices and products, experiments or obsolete electronic devices, both DIYbio hackers and Obsoletos try to break and revert certain broadly imposed limits and obstacles: the timing, agenda, methods, instruments and topics of an entire discipline, or the obsolete durability and lifespan of electronic designs. DIYbio and e-waste hacking is thus a way of questioning closures and precarious material conditions produced by post-industrial capitalism in the age of the Internet. This resonates with Dewey’s notion of the inquiry, we argue in this section.

The work of John Dewey (1922, 1927) introduces an experimental approach to politics and education. In this empiricist and pragmatic account, the ‘inquiry’ is a key practice in which situations are problematised and disclosed as undetermined, which are then given a determination and a solution. The imagination is a key element in Dewey’s concept of inquiry: it makes it possible to see the actual in light of the possible.^9^ In Dewey’s (1938) words, inquiry is defined as

The controlled or directed transformation of an indeterminate situation into one that is so determinate in its constituent distinctions and relations as to convert the elements of the original situation into a unified whole […] The original indeterminate situation is not only ‘open’ to inquiry, but it is open in the sense that its constituents do not hang together.

That things do not ‘hang together’ or do not necessarily need to in the way they do at present is what hacks ultimately show, as our empirical sections suggest. In different ways, DIYbio and e-waste hacks work as tangible proof that realities are not finished or given but that they are sites for inquiry. As in Obsoletos’ ChipBot or in the BioStrike project, hacks disclose problematic situations as undetermined and modifiable and then turn them into an opportunity for collective inquiry. Differing from Dewey’s account though, in DIY and makers’ practices, the site where the inquiry takes place is particular objects. The point of departure are tools accessible on the Internet, such as discarded lab equipment and materials within reach, such as kitchen equipment or old computers. These objects are often mundane, remnants of the surplus of knowledge- and consumer-based societies. It is by creatively reusing and modifying them in the process of inquiry, as well as by demonstrating their material precariousness and taking advantage of it, that problematic situations, such as the monopolies and unsustainable consumption patterns and management processes of waste, are revealed as problems that can be ‘coped’ with.

However, as we have seen, in DIYbio and e-waste, hacks are not necessarily produced to solve problems. Rather, hacks are experiments: they show that problems can be solved and that things could *be done otherwise*. In this experimental mode of inquiry, the emphasis is not necessarily placed on whether hacks actually work but on the work that goes into trying to make them work. By tinkering – that is, trying things and trying them again, perhaps in an unconventional way – there lies the exploratory and fun dimension of hacking. Arguably, Dewey’s notion of inquiry misses this element because it is often presented as a quite intellectual activity, often reserved to experts.^10^ When e-waste hackers create a bubble-maker from a CD reader and biohackers make broth to feed their microbes or test their own bodies, they perform a different way of using electronics and biology (through doing) and question the process of the inquiry itself by bringing it to the terrain of the immediate and mundane. What is hacked is not only a site that was inaccessible before (i.e. biology or electronics) but also the way of accessing it. By exposing themselves as easy and unsettled, hacks work as tangible demonstrations that ‘*you can also do it*’. In doing so, such demonstrations are not only technical but also political.

DIYbio and e-waste hacks as inquiry ensure their continuity by ensuring access, as well as by inviting further experimentation. This is achieved not only by using accessible materials but also by carefully documenting how the hack was done, even if the experiment did not go ‘well’. The crucial matter is that the experiment itself is given^11^ to others to replicate and modify, so that it might be further developed. The work of documenting is decisive for ensuring this constant circulation of knowledge. The Internet works as a shared repository for the collective imagination. If someone wants to hack something, probably someone has done it before or someone has a good idea of how to do it, as Obsoletos remarks. The possibility of individual hacks depends on the collective work of documenting and sharing, as Gabriella Coleman (2012) showed in her ethnographic work on hacking. Sharing practices generates a recursive movement in which hackers ensure the possibility of their own continuity as both individuals and collectives.^12^ Furthermore, sharing (by documenting) on the Internet, as DIYbio and e-waste hackers do, produces a technical visibility that turns potentially *everyone (anyone)* into a public, a virtual witness (with potential to replicate the hack any time, any place). The public display triggered by sharing practices opens up the possibilities of action in an attempt to attract new potential hackers. In this performativity, hacks enable the proliferation and traffic of particular and situated appropriations that return and flow back in a more open and public stream (Gauntlett, 2011; Meyer, 2013). Alongside, much work also goes into organising offline events, such as workshops and courses in which simple hacks are publicly performed. These events show that hacking biology and electronics is ‘easy’. The deliberate emphasis on ‘easiness’ may serve as a resource to connect with and attract other people (beyond the usual community of geeks).

By sharing, documenting and showing how easy hacking is, DIYbio and e-waste hackers create a multi-dimensional space of visibility, showing to others that realities such as life and waste are ontologically precarious (they change and can be turned into something else), epistemologically undetermined (can always be modified) and politically accessible. Hacks are not presented as ultimate truths or definitive solutions, but as prototypes. As such, they are shown as technically open-ended and unfinished, enabling further (collective) action, but also questioning established models of science and society (Corsín, 2013).^13^

Seen as prototypes, hacks enable a type of *politics of demonstration*^14^ that relies not on the production and display of evidence, but on the production and display of a precarious relationality. As in Dewey’s account of the inquiry, hacks show that things could possibly hold together in a different way. In a complementary approach, Judith Butler (2004) identifies precariousness as an ontological condition, and the collective acknowledgement of it as a condition of possibility for collective and public action. Precarity, on the other hand, is a state in which political agencies (or the State) do not produce security (hence coping with the ontological precariousness of human lives); rather, it induces precariousness. Precarity is the state that current capitalist politics produce, a state of increasing dependency and vulnerability (Butler, 2009). Hence, public action, according to Butler (2012) and also closely connected with Dewey’s notion of the public, arises when people gather together to rally against ‘induced conditions of precariousness’ (p. 14). Up to this point, we have argued that DIY and makers’ practices are a product and a response to the current ‘state of things’. In hacking, precarious objects are produced to cope with the precarity of times. They can be seen as political demonstrations. By showing ontological precariousness, they propose a different way of relating to technology and a particular form of sociality (Tocchetti, 2012), one in which control over technology is not delegated but rather ‘owned’ in an attempt at gaining control over one’s own life conditions. And because it is ‘owned’, it can also be shared, potentially enabling further collectives and new ways of being connected through experiencing technology. Showing that *you can do it*, DIY and makers’ hacks mobilise political action by questioning dependencies on institutions that do not serve to cope with the precarious condition of life but rather induce precarity through an economy of overproductions and obsolescence. To this point, the ambiguity is as follows: while the overproduction and obsolescence inscribed in consumer and knowledge-based societies is contested in the proliferation of hacks, DIYbio and e-waste hacks also depend on that overproduction and obsolescence.

## 5. Final notes: From the politics of representation to a politics of demonstration

In this article, we have argued that DIY and makers’ hacks can be seen as a sort of political action that entails a move towards a politics of demonstration. Hacks can be seen as a collective form of inquiry different from that proposed by Dewey: hacks neither necessarily provide defined solutions for problems, nor are they particularly concerned with producing evidence. Rather, DIY and makers’ hacks display issues as public by making precarious objects. By playing on the precariousness of technological and living entities, DIYbio and e-waste hacks show that *you* can also *do it*. Such hacks are a call for immediate and direct action that challenges Dewey’s account of the articulation of issues: how issues are ultimately turned into problems to be addressed by State authorities. This sort of ‘bureaucratisation’ of the issue is key in Dewey’s (1927) account of politics.^15^ However, DIY hacks in a sense suspend this second moment of the articulation of issues as public. As Obsoletos hackers remark, they do not aim to ‘preach’ but to raise visibility over certain common problems. What is crucial, our analysis has shown, is how that visibility is worked out through practices of sharing and collective circulations. Hacks are tangible demonstrations that realities could hold together differently and potentially, anyone could modify them.

Arguably, performed as public demonstrations, hacks are ‘mobilisation apparatuses’ (Rosental, 2013). In the technical visibility that on and offline sharing practices enable, hacks are made to have a ‘contaminating’ or viral effect. They deploy a sort of direct action that can perform as ‘an example that others can imitate’ (Graeber, 2009: 211; see also McKay, 1998). In this sort of potential virulence, DIY and makers’ hacking might appear as imposing new technical demands on citizens, pointing to a sort of ‘techno-citizenship’ in which technical literacy (or even autonomy) might be expected. Citizens may in this way appear as technology developers that must take control or even responsibility (Rosenberg, 2011) for their own precarious life conditions.

Finally, by hacking the material world around and by making precarious objects, DIYers and makers propose a way to cope with the increasing precarisation of citizens’ life conditions and rights. This is already having politicising and empowering effects (Graham and Thrift, 2007; Jackson, 2013), and it may further prompt formation of political collectives and articulations. However, within DIY and makers’ politics of demonstration, the line between coping with ontological precariousness and accepting political precarity becomes blurry. A danger of such acceptance is a transfer of responsibilities previously attributed to the State to the citizenry in basic realms of everyday life, such as health and waste management. Another potential problem of portraying citizens as problem solvers is the possible outsourcing (performed as crowdsourcing) of ideas and work force from the public to industry under the umbrella of social innovation. If the role of active technology developers is to be demanded from citizens, a question remains, ‘What new set of civic rights and institutions should accompany the new technological duties?’

## Notes

1. Descriptions on do-it-yourself biology (DIYbio) are based on fieldwork observations carried out in different European cities since 2011. This includes observations (mainly in workshops), informal talks and semi-structured interviews with DIYbio members, observation of online forums, blogs, wikis and email lists among other online sources and active participation in discussions with DIYbio practitioners (mainly on biocommons and ethics and also on education and politics) within the framework of the Mobilization and Mutual Learning Action ‘SYNENERGENE’ (funded by the European Commission). The fieldwork on electronic waste (e-waste) is based on Obsoletos.org project (Madrid). During October and November 2012, participatory observation was carried on in La Nave, their workplace, together with a series of collective interviews and analysis of Obsoletos’ blogsite (their main resource for communicating and documenting their activities).

2. Other authors might have observed differing trends in other parts of the world. For instance, scholars have reported on the marked corporative character of DIYbio groups in the United States (Delfanti, 2014; Grunshkin et al., 2013). Our arguments on the public dimensions of DIYbio activities are restricted to the author’s observations in Europe.

3. J. Butler (2004) introduces a distinction between ontological precariousness and political precarity – see Section 4 in this article.

4. The first author of this article participated in the organisation of this event. After this workshop, the participants were asked to evaluate it.

5. Documentation about the BioStrike project can be found at: https://brmlab.cz/project/biolab/biostrike.

6. Bioprospecting refers to massive screening for the identification of active biocompounds with commercial interest. It has been often used in the context of indigenous knowledge (whose knowledge would serve to such screening) and biodiversity conservation.

7.
http://www.bento.bio/

8. Referring to ‘resources’, in this instance ‘electronic waste’.

9. As Thomas Alexander (1993) explains in his work about Dewey and the Moral Imagination, imagination was
a creative exploration of structures inherited form past experiences which thereby allowed the futures as a horizon of possible actions, and so of possible meanings to guide and interpret the present. […] It was neither mere copying nor radical creation ex nihilo [but] the transformation or reconstruction of experience in a changing world which nevertheless admitted general stable features. (p. 183)

10. See the last chapter of ‘The Public and Its Problems’, on the inquiry.

11. Or as a ‘gift’ in Marcel Mauss’ (2000 [1950]) sense, producing an expectation of reciprocity on the other side.

12. Chris Kelty (2008) documented these dynamics in detail in his ethnographic work on hackers and geeks. Kelty coins the figure of the ‘recursive public’. As they maintain and share technology and infrastructures, DIY and e-waste can also appear as both publics and catalysers of new publics.

13. The work of prototyping, as Corsín (2013: 6) suggests, employs such open-endedness to deliberate political effects for the continuous re-emergence of undetermined events that summon their own openness to future tinkering, where relationships and objects are at once ‘more than many and less than one’.

14. In the context of the politics of women’s bodies, Murphy (2004) described a similar type of visibility produced by alternative ways of using a particular object (the spectrometer, used for vaginal self-examination).

15. ‘The lasting, extensive and serious consequences of associated activity bring into existence a public. In itself it is unorganized and formless. By means of officials and their special powers it becomes a state’ (Dewey, 1927: 67).
